# Fast-starting after a breath: air-breathing motions are kinematically similar to escape responses in the catfish *Hoplosternum littorale*

**DOI:** 10.1242/bio.20149332

**Published:** 2014-12-19

**Authors:** Paolo Domenici, Tommy Norin, Peter G. Bushnell, Jacob L. Johansen, Peter Vilhelm Skov, Morten B. S. Svendsen, John F. Steffensen, Augusto S. Abe

**Affiliations:** 1 IAMC-CNR Oristano, Loc. Sa Mardini, 09170 Torregrande (OR), Italy; 2 Zoophysiology, Department of Bioscience, Aarhus University, DK-8000 Aarhus C, Denmark; 3 Department of Biological Sciences, Indiana University South Bend, South Bend, IN 46634, USA; 4 ARC Centre of Excellence for Coral Reef Studies, James Cook University, Townsville, QLD 4811, Australia; 5 School of Marine and Tropical Biology, College of Marine and Environmental Sciences, James Cook University, Townsville, QLD 4811, Australia; 6 DTU Aqua, Section for Aquaculture, Technical University of Denmark, The North Sea Research Centre, P.O. Box 101, DK-9850 Hirtshals, Denmark; 7 Marine Biological Laboratory, University of Copenhagen, Strandpromenaden 5, DK-3000 Helsingør, Denmark; 8 Department of Zoology, University of São Paulo State, 13506-900 Rio Claro, SP, Brazil; * Present address: Ocean Sciences Centre, Memorial University of Newfoundland, St. John's, NL A1C 5S7, Canada.; ‡ Present address: Whitney Laboratory for Marine Bioscience, University of Florida, St. Augustine, FL 32080, USA.

**Keywords:** Air-breathing, Behaviour, C-start, Escape response, Fish, Kinematics

## Abstract

Fast-starts are brief accelerations commonly observed in fish within the context of predator–prey interactions. In typical C-start escape responses, fish react to a threatening stimulus by bending their body into a C-shape during the first muscle contraction (i.e. stage 1) which provides a sudden acceleration away from the stimulus. Recently, similar C-starts have been recorded in fish aiming at a prey. Little is known about C-starts outside the context of predator–prey interactions, though recent work has shown that escape response can also be induced by high temperature. Here, we test the hypothesis that air-breathing fish may use C-starts in the context of gulping air at the surface. *Hoplosternum littorale* is an air-breathing freshwater catfish found in South America. Field video observations reveal that their air-breathing behaviour consists of air-gulping at the surface, followed by a fast turn which re-directs the fish towards the bottom. Using high-speed video in the laboratory, we compared the kinematics of the turn immediately following air-gulping performed by *H. littorale* in normoxia with those of mechanically-triggered C-start escape responses and with routine (i.e. spontaneous) turns. Our results show that air-breathing events overlap considerably with escape responses with a large stage 1 angle in terms of turning rates, distance covered and the relationship between these rates. Therefore, these two behaviours can be considered kinematically comparable, suggesting that air-breathing in this species is followed by escape-like C-start motions, presumably to minimise time at the surface and exposure to avian predators. These findings show that C-starts can occur in a variety of contexts in which fish may need to get away from areas of potential danger.

## INTRODUCTION

Fast-starts are brief accelerations commonly observed in fish within the context of predator–prey interactions (Domenici and Blake, 1997). Both predator strikes and the escape responses of prey are considered fast-starts, and these have been studied from many perspectives, including biomechanics, muscle physiology, neurobiology and behaviour (Domenici and Blake, 1997; Korn and Faber, 2005; Wakeling, 2006). During escape responses, fish typically respond to a threatening stimulus with a unilateral contraction of their axial muscle (stage 1) that results in bending their body into a C-shape directed away from the threat, which may be followed by a contralateral contraction (stage 2) (Domenici and Blake, 1991; Domenici and Blake, 1997). These C-start escape responses are usually controlled by one of a pair of giant reticulospinal neurons, the Mauthner cells (for a review, see Korn and Faber, 2005).

In recent years, a large body of evidence has highlighted the occurrence of many “variants” of the escape response, including responses lacking a stage 2 (single-bend responses) (Domenici and Blake, 1991; Lefrancois et al., 2005), S-start responses in which contractions on both sides of the body occur during stage 1 (Hale, 2002), and responses that are not controlled by the Mauthner cells but rather by alternative neural circuits which generate lower performance and slower reaction times (Eaton et al., 2001; Kohashi and Oda, 2008). The flexibility of the fast-start system is further illustrated by the presence of a number of behaviours that fish accomplish using escape-like C-start motions, i.e. motions that are kinematically similar to C-start escape responses but that are observed outside the context of responding to a predator attack. Examples of these C-starts include the fast body turns observed in cichlids (*Astatotilapia burtoni*) during agonistic displays in the presence of conspecifics (Fernald, 1975), the post-feeding turns made by goldfish (*Carassius auratus*) after having captured a prey item on the surface (Canfield and Rose, 1993), the C-start strikes of archerfish (*Toxotes jaculatrix*) and fruit-catching fish (*Brycon guatemalensis*) towards prey or food items fallen on the water surface (Wöhl and Schuster, 2007; Krupczynski and Schuster, 2008), and the C-start in goldfish striking an object which may have evoked an innate “prey strike” behaviour (Canfield, 2007). Overall, most of these C-start behaviours studied thus far have been related to interactions with predators, prey items or conspecifics, and always in relation to direct external stimuli. Interestingly, recent work has shown that escape-like turns may even be triggered by abiotic factors, such as high temperature (Sillar and Robertson, 2009). In this case, the escape-like turns observed in tadpoles (*Xenopus laevis*) may be a means of avoiding potentially damaging high temperatures (Sillar and Robertson, 2009). Therefore, animals such as fish and tadpoles may use escape-like turns more widely than just for the typical startle response triggered by a startling sudden stimulation, which has been the main approach in studies on animal escape responses (Domenici and Blake, 1997).

There are potentially many instances in which fish may need to execute a behaviour quickly, as it can be done with a C-start. Among these, air-breathing in fish often requires that individuals minimise the time spent gulping air at the surface where they would make themselves visible and accessible to predators (Kramer et al., 1983). Indeed, in the presence of a model predator, air-breathing fish, as well as those performing aquatic surface respiration, tend to decrease the frequency of surfacing (Kramer, 1987; Shingles et al., 2005).

The catfish *Hoplosternum littorale* (Hancock) is a facultative air-breather that lives in rivers and ponds in South America. Because of its air-breathing behaviour, *H. littorale* can easily cope with hypoxia (Sloman et al., 2009) but this species also takes air from the water surface during normoxia (Affonso and Rantin, 2005). Field observations made at natural ponds in the Pantanal wetlands of Mato Grosso do Sul, Brazil, suggest that the turns made by *H. littorale* in relation to air-breathing are performed rapidly with a sharp bending of the body after contact with the water surface. Using both laboratory and field observations, we therefore used this species to test the hypothesis that the turn that follows immediately after air-gulping, re-directing the fish towards the bottom, is kinematically similar to a typical escape response triggered by mechanical stimulation, thereby demonstrating that escape-like C-start motions can be used in the contexts of air-breathing in the absence of predators or prey.

## MATERIALS AND METHODS

### Animals and holding conditions

Wild *H. littorale* were caught over three days in mid October 2011 by cast netting in ponds near Rio Claro (São Paulo State, Brazil) and transported by road to the Department of Zoology, University of São Paulo State, Rio Claro, São Paulo, Brazil. Here, fish were kept in the laboratory in rectangular tanks, each containing approximately 70 L of fully aerated, dechlorinated tapwater at 24–26°C. Fish were kept in the laboratory for 2–3 days without feeding before experiments were conducted. A total of 70 individual fish were used (total length  =  12.8±0.1 cm; body mass  =  31.6±0.7 g; mean ± SE) in 70 trials separated into 38 escape responses, 16 air-breathing events and 16 routine turns. Each individual fish was used only once.

All procedures were approved by a local ethical review committee and conformed to the relevant regulatory standards.

### Air-breathing setup

Recordings of air-breathing events (ABE) were made by transferring individual fish, chosen haphazardly from their holding tanks, into a 90 L glass aquarium (60 × 30 × 50 cm; length × width × height) containing approximately 60 L of fully aerated, dechlorinated tapwater at 24–26°C. The aquarium was divided longitudinally with an opaque white plastic screen to ensure that the fish stayed within the depth of field for video recordings. 5 × 5 cm square markings on the screen served as a reference for later analyses. This setup allowed the fish to move freely within a section of the aquarium (52 × 15 × 33 cm; length × width × water depth) where high-speed recordings of voluntary ABE were obtained at 240 frames s^−1^ using a Casio Exilim FH100 digital camera placed 2.7 m from the aquarium. A mirror placed at an angle of 45 degrees next to the tank allowed lateral view of the event. The portion of ABE analysed consisted of the fast turn made by the fish after contact with the water surface. The sequence of events before and after ABE was: (a) fish left the bottom of the tank and swam towards the water surface approximately perpendicular to it, (b) the fish made contact with the water surface, (c) the fish made a quick turn (i.e. recorded as ABE), and (d) the fish swam back towards the bottom of the tank (also see supplementary material Fig. S1). Since kinematic 2D analyses of air-breaths required swimming motion during ABE to be perpendicular to the camera as judged through the mirror, 3–5 air-breaths were recorded for each fish and one was chosen for analysis.

### Escape response setup

The setup for recordings of escape responses (ER) consisted of a 140 L white rectangular experimental tank (70 × 50 × 40 cm; l × w × h) containing approximately 42 L of fully aerated, dechlorinated tapwater at 24–26°C (water depth  =  12 cm). Fish were chosen haphazardly from their holding tanks and individually introduced to the experimental tank where they were allowed a minimum of 30 min to settle before experiments were started.

An ER was elicited by dropping a truncated rubber cone (the stimulus) weighing 48 g (4.3 × 2.2 × 4.0 cm; bottom d × h × top d) onto the water surface through a 75 cm long, grey pipe (10 cm diameter) hanging vertically over one end of the experimental tank and with the bottom edge at a distance of 1 cm above the water surface. The centre of the pipe was positioned 25 cm from the near-end and side walls of the tank, and 45 cm from the far-end of the tank (supplementary material Fig. S2). A string attached to the stimulus allowed the experimenter to control the timing of release remotely and prevented the stimulus from hitting the bottom of the tank. A webcam positioned above the tank showed the position of the fish on a laptop, allowing the experimenter to visually monitor the experimental arena while staying out of sight of the fish. An escape response was elicited by dropping the stimulus when the fish entered the drop zone (i.e. came within 15 cm from the impact zone beneath the pipe; supplementary material Fig. S2). Fish were recorded while motionless or gliding at very low speed (<0.5 body length s^−1^). A Casio Exilim FH100 digital camera was mounted 80 cm above the water surface and recorded the escape at 240 frames s^−1^. A 30 cm ruler placed on the bottom of the tank served as a reference for later analyses. By simultaneously filming a mirror on the tank wall, showing the water surface below the pipe, the timing of escape could be coupled with the impact of the stimulus onto the water surface, allowing for calculations of escape latency.

### Routine turn setup

Using the same tank as the one for escape responses, routine turns (RT) (i.e., the fish turning spontaneously without being startled) of individual fish were recorded. Fish were left undisturbed in the tank for a minimum of 30 minutes before recording. Recordings were carried out using the same camera set-up and the same frame rate as in the escape response trials.

### Field observations

Field observations of ABE (field-ABE; N = 54) were carried out in a natural pond (approximately 40 m in diameter) in the Pantanal, Mato Grosso do Sul, Brazil (19°31′33″S, 57°02′27″W). Water temperature and oxygen levels were measured in the pond during the daytime field observations using a portable CTD (conductivity, temperature and depth instrument). Water temperature and oxygen levels were between 29.5 and 29.8°C and 94 and 151% air saturation, respectively, at 0.5 to 1 m depth. Overnight measurements showed that water temperature could range between ∼26°C in the early morning (before sunrise) to ∼35°C in the afternoon, with oxygen levels ranging from anoxic (0% air saturation) during most of the night to super-saturated (as high as 270% air saturation) around midday. *H. littorale* were filmed from the surface using a high-speed video camera (Casio Exilim FH100) at 240 frames s^−1^. Videos were taken from a bridge approximately three meters above the water level. Duration of the C-bends (i.e., stage 1 duration) occurring during air-breathing were estimated from the videos, and the turning rates of the turn that occurred immediately after contact with the water surface were calculated using the average stage 1 angle observed in the laboratory (136.3 degrees). However, in order to make a more conservative estimate and test if the use of alternative average stage 1 angles affected our results, we have also used the minimal angle a fish would require in order to dive back into the water after emerging perpendicularly to the surface. This corresponds to an angle slightly larger than 90 degrees (otherwise the fish would be skimming at the surface), such as 100 degrees. Indeed, observations in the laboratory show a minimum S1A of 103 degrees (see Results). The sizes of fish in this pond was estimated to be similar to that used in the laboratory (i.e. total length  =  12–13 cm), which is typical for adult fish of this species. Although these mean turning rates could only be estimated, due to uncertainties in fish size and limited precision in the determination of stage 1 duration, they should nevertheless provide an indication of the swiftness of the air-breathing events in field situations.

### Data analysis

Data were analysed in terms of kinematic performance and timing of the response using video analysis software (WINanalyze; Mikromak, Germany) to digitise the center of mass (CM) and the tip of the snout frame by frame. For air-breathing events (ABE), the fast turns the fish made after gulping air at the surface were analysed. The CM was determined following Domenici and Blake (Domenici and Blake, 1991) to be at a distance of 0.35 body length (0.35±0.003 body lengths; N = 3) from the tip of the snout using 3 dead specimens.

Stage 1 angle (S1A) was defined as the angle between the line joining the CM and the snout at the beginning of the response and the same line at the end of the turn accomplished during the first body bend (i.e. stage 1 of the escape response and of the air-breathing manoeuvre). The end of stage 1 was defined as the reversal of the turning direction of the head (Domenici and Blake, 1997). Stage 1 duration (S1D) was defined as the time taken to accomplish stage 1 in both escape and air-breathing manoeuvres. Mean turning rate (TR_mean_) was calculated by dividing S1A by S1D. Maximum turning rate (TR_max_) was calculated as the maximum angular velocity of the line passing through the snout and the CM during stage 1. The distance between the CM of the fish at the frame before the first visible turning response and 100 ms later was used to define the distance travelled in 100 ms (D100). This variable was measured only in ER and ABE. Escape latencies were measured as the time from contact between the stimulus and the water, to the first visible reaction of the fish. Escape latencies were measured only in escape responses.

Non-parametric ANOVA (Kruskal–Wallis) was used to compare data among ER, ABE and RT, since, for each variable, at least one of the groups showed a non-normal distribution (Kolmogorov–Smirnov test). Post-hoc test (Dunn's) was used to compare data between two groups. The comparison of TR_mean_ also included field-ABE.

## RESULTS

Our observations of wild-caught *H. littorale* performing air-breathing in the laboratory showed that the fish typically approached the water surface swimming slowly up from the bottom. Once the fish made contact with the surface, they performed a fast turn away from the surface which re-directed them towards the bottom of the tank (see supplementary material Fig. S1 for a frame-by-frame illustration of a post-air-gulping turn).

### Stage 1 angle (S1A) and stage 1 duration (S1D)

**Fig. 1.**
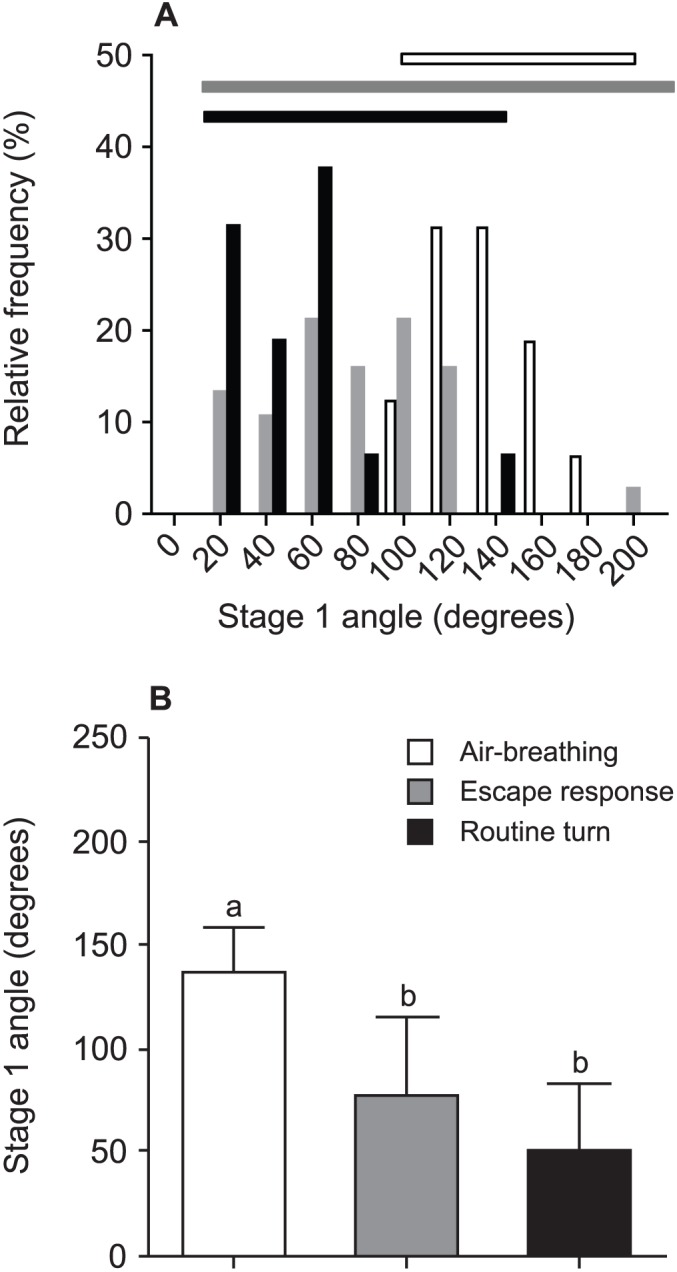
Stage 1 angle (S1A) of air-breathing events (white bars; N = 16), escape responses (grey bars; N = 38) and routine turns (black bars; N = 16) for *Hoplosternum littorale*. (A) The relative frequency of S1A for the three groups in 20 degree bins with horizontal lines representing the span of S1A for each group. (B) S1A (mean ± SE) for the three groups. Different lower-case letters above bars denote significant differences between groups.

For air-breathing events (ABE) and escape responses (ER), there was a clear overlap in the range of S1A (Fig. 1A). However, ABE were more limited in range (103–182 degrees), relative to the >180 degrees span of ER (11–194 degrees). S1A were statistically different among the three groups of ABE (mean ± SE  =  136.3±5.5 degrees), ER (mean 77.2±6.2 degrees) and routine turns (RT; mean 51.5±8.1 degrees) (Kruskal–Wallis, P<0.001; Fig. 1B). Post-hoc tests revealed that S1A for ABE was significantly greater than for ER and RT (P<0.05 in both cases), but no differences were found between ER and RT (P>0.05). S1D differed among the three groups (Kruskal–Wallis, P<0.05). Post-hoc tests showed that S1D in RT (range 150–550 ms; mean 225.7±24.5 ms) and in ABE (range 58.3–200 ms; mean 127.1±10.0 ms) were significantly longer than in ER (range 20.8–108.3; mean 51.6±3.0 ms) (P<0.05 in both cases), while no differences were found between the S1D in ABE and RT (P>0.05).

### Turning rates (TR)

**Fig. 2.**
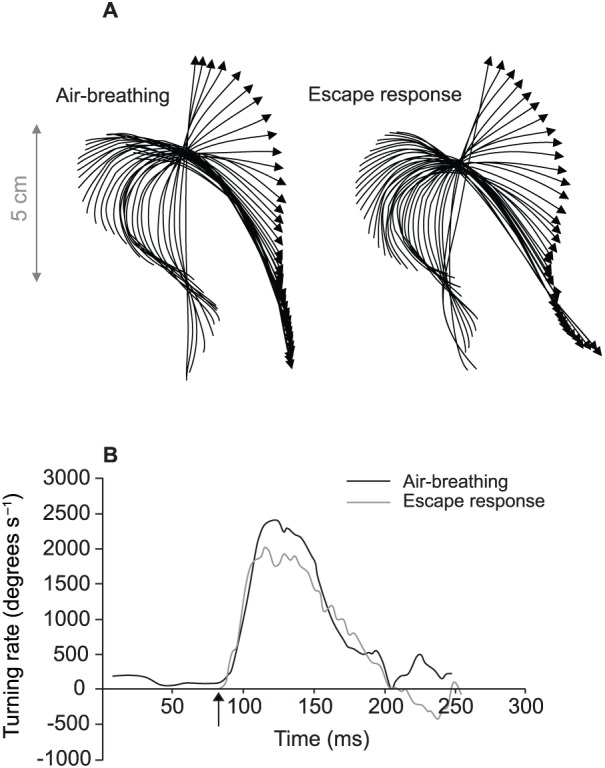
Example of an air-breathing event (ABE) and an escape response (ER) with similar S1A for two *Hoplosternum littorale*. (A) Tracings of the mid-line of the fish with arrow heads indicating the position of the snout of the fish while the other end of the arrow represents the tail of the fish. Each line is separated in time by 4.17 ms (i.e. frame-by-frame at 240 frames s^−1^). (B) The time course of turning rates, with stage 1 starting at the position of the vertical arrow and coming to an end where the lines touch the *x*-axis at ∼205 ms.

Fig. 2A shows examples of the midline of the fish in events with a similar S1A and Fig. 2B shows the respective time course of the turning rates. In these examples, the turning rates overlap considerably.

**Fig. 3.**
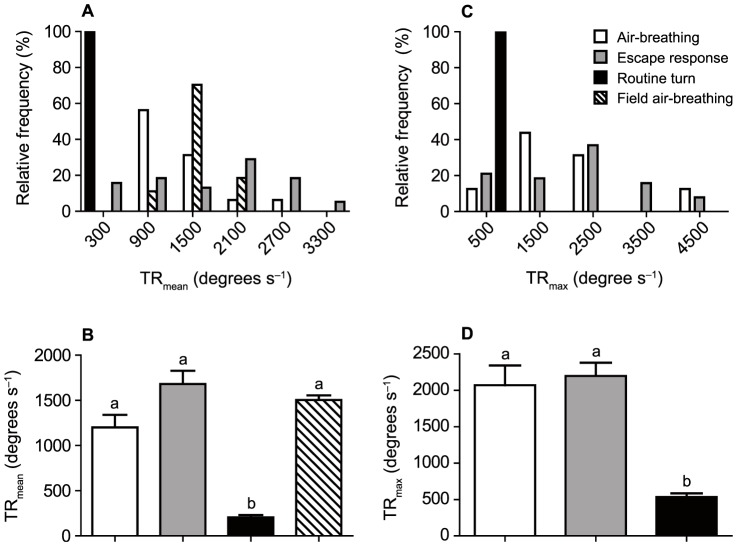
Turning rates of *Hoplosternum littorale* for air-breathing events (ABE, white bars; N = 16), escape responses (ER, grey bars; N = 16), routine turns (RT, black bars; N = 16) and field air-breathing events (field-ABE, striped bars; N = 54). Note that turning rates of field-ABE were estimated from the mean S1A observed in the laboratory (see Materials and Methods), hence maximum turning rates were not calculated. (A) Relative frequencies of mean turning rate (TR_mean_) show a considerable distribution and overlap of ABE, ER and field-ABE, whereas RT are generally performed within a narrow range; bin size is 600 degrees. (B) Overall means ± SE for TR_mean_ show that RT are performed at a significantly lower mean rate than either ABE, ER or field-ABE, with different lower-case letters denoting significant differences between groups. (C) Similar to TR_mean_, relative frequencies of maximum turning rate (TR_max_) show a considerable distribution and overlap of ABE and ER, whereas RT are generally performed within a narrow range; bin size is 1000 degrees. (D) Overall means ± SE for TR_max_ show that RT are also performed at a significantly lower maximum rate than either ABE or ER, with different lower-case letters denoting significant differences between groups.

The values of TR_mean_ ranged between 647–2526 degrees s^–1^ (mean 1200±126.4 degrees s^–1^), 221–3395 degrees s^–1^ (mean 1681±146.5 degrees s^–1^) and 53–416 degrees s^–1^ (mean 205.5±24.9 degrees s^–1^) for ABE, ER and RT, respectively (Fig. 3A). Estimated TR_mean_ of field-ABE ranged 1128–2181 degrees s^–1^ (mean 1504±36.7 degrees s^–1^) (Fig. 3A). TR_mean_ were statistically different among the four groups (ER, RT, ABE and field-ABE) (Kruskal–Wallis, P<0.0001; Fig. 3B). Post-hoc tests revealed that the TR_mean_ of ER, ABE and field-ABE were all significantly higher than that of RT (P<0.05 in all three cases), while no differences were found when comparing ABE and ER, ER and field-ABE, and ABE and field-ABE (all P>0.05). If a more conservative estimate of S1A (i.e. 100 degrees, see Materials and Methods) is used, the values of TR_mean_ for field-ABE show a mean of 1103±26.9 degrees s^–1^ (range 828–1600 degrees s^–1^). However, even using this conservative estimate, the results of the Kruskal–Wallis test is still significant (P<0.0001) and the post-hoc tests still show that TR_mean_ of ER, ABE and field-ABE were all significantly higher than that of RT (P<0.05 in all three cases), while no differences were found when comparing ABE and ER, ER and field-ABE, and ABE and field-ABE (all P>0.05).

TR_max_ ranged between 942–4840 degrees s^–1^ (mean 2070±270.7 degrees s^–1^), 380–4215 degrees s^–1^ (mean 2196±180.9 degrees s^–1^) and 182–826 degrees s^–1^ (mean 533.3±50.2 degrees s^–1^) for ABE, ER and RT, respectively (Fig. 3C). TR_max_ were statistically different among ABE, ER and RT (Kruskal–Wallis, P<0.0001; Fig. 3D). Post-hoc tests showed that both ER and ABE had a significantly higher TR_max_ than RT (P<0.05 in both cases), while no differences were found between ABE and ER (P>0.05).

### Distance travelled in 100 ms (D100)

The values for D100 ranged 2.1–57.8 mm (mean 22.2±4.5 mm), 3.2–105.0 mm (mean 35.5±3.5 mm) and 1.5–11.7 mm (mean 4.9±0.8 mm) for ABE, ER and RT, respectively. D100 was statistically different between ER, ABE and RT (Kruskal–Wallis, P<0.0001). Post-hoc tests showed that D100 in ER and ABE was significantly greater than in RT (P<0.05 in both cases), while no differences were found between ER and ABE (P>0.05).

### Relationship between turning rate and distance

**Fig. 4.**
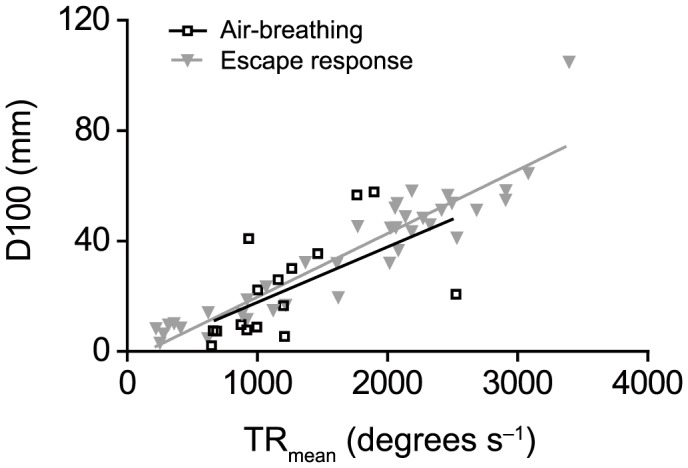
Correlations between distance travelled in 100 ms (D100) and mean turning rate (TR_mean_) for air-breathing events (ABE, squares) and escape responses (ER, triangles). Linear regression equations are *y* = 0.02*x*–2.39 (r^2^ = 0.34; P<0.05; N = 16) and *y* = 0.02*x*–3.31 (r^2^ = 0.87; P<0.001; N = 38) for ABE and ER, respectively. There were no significant differences between either slope or elevation of the two lines.

A linear, positive relationship between TR_mean_ and D100 was found to be significant for both ABE (r^2^ = 0.34; P<0.05; N = 16) and ER (r^2^ = 0.87; P<0.001; N = 38), and the two slopes and elevations were not significantly different from each other (ANCOVA; P>0.05 for both slope and elevation; Fig. 4).

### Relationship between turning rate and latency

**Fig. 5.**
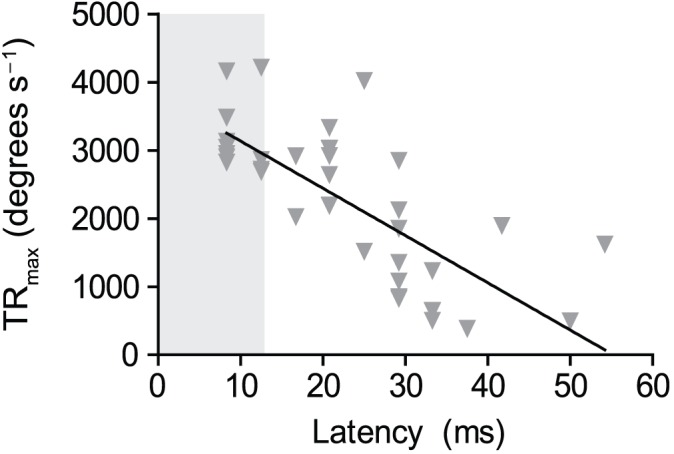
Relationship between maximum turning rate and latency of escape responses (*y* = −69*x*+3829; r^2^ = 0.55; P<0.001; N = 38). The shaded area covering latencies ≤12.5 ms indicate escapes that are likely to be controlled by the Mauthner cells (see Results for further explanation).

A significant negative relationship between TR_max_ in ER and their escape latency (i.e. time from stimulation to first movement of the head) was found (r^2^ = 0.55; P<0.0001; N = 38; Fig. 5). In the present study, ER latencies ranged from 8.3 to 54.2 ms, with 29% being ≤12.5 ms. If we consider that the shortest latencies (≤12.5 ms) are likely to be Mauthner cell controlled (Eaton et al., 2001), these show a TR_max_ of 2686–4215 degrees s^−1^. The range of TR_max_ in these short-latency ER largely overlaps with that of ABE (942–4840 degrees s^−1^). Hence, while latencies in ABE could not be measured, the range of TR_max_ in ABE overlaps with the TR_max_ of the ER that are most likely to be Mauthner-cell driven [fastest TR and shortest latencies (Liu and Fetcho, 1999; Kohashi and Oda, 2008)].

## DISCUSSION

Our findings demonstrate that C-starts in fish can be used within the context of air-breathing and provide a novel comparison of the kinematics and performance of an air-breathing manoeuvre with that of escape responses within a species. The results show that air-breathing events (ABE) performed by *Hoplosternum littorale* are kinematically similar to C-start escape responses (ER), as mean performance levels of these behaviours do not differ and the range of values largely overlap in terms of turning rates, distance covered and the relationship between these rates. Although the ranges of turning angles in ABE and ER also overlap, there are significant differences between the means. The higher mean stage 1 angle (S1A) of ABE compared to ER (136.3 *vs.* 77.2 degrees, respectively) can, however, be easily explained by the need to dive quickly back towards the bottom after a vertical ascent towards the water surface, which is reflected in the limited range of S1A observed in ABE (103–182 degrees) compared to the wide range of S1A observed in ER (11–194 degrees) typical of fish escape responses (Domenici and Blake, 1997). While significant differences in the mean values can imply that the two behaviours accomplish different tasks, large overlaps in performance values would suggest that the physiological mechanisms driving the two responses are similar [see also Wöhl and Schuster (Wöhl and Schuster, 2007) and Canfield (Canfield, 2007) for comparisons of escape-like behaviours based on performance overlaps]. Indeed, when considering only ER with large angles (within the range of S1A observed in ABE, i.e. 103–182 degrees), the overlap in performance with ABE is considerable (67%, 100% and 67% of the ER values are within the range of ABE values observed for TR_mean_, TR_max_ and D100, respectively). ABE may therefore be considered kinematically comparable to a subset of ER, i.e. those with a large S1A. Interestingly, routine turns show a limited range of S1A with most turns <90 degrees, possibly because large turns are not necessary for exploring the environment.

Although ABE and ER show largely overlapping performance levels, it is possible that certain differences in the starting position may have affected the performance of the two behaviours differently. For example, ABE were performed vertically, near the surface and while the fish was in motion while ER were performed in the horizontal plane, away from the surface and while the fish was motionless or gliding slowly. Prior to ABE, fish typically stopped their forward motion once the fish made contact with the surface; hence the starting speed of both ER and ABE was in all cases negligible. However, ABE and ER may differ in terms of both surface and ground effects (Blake, 1983). Nevertheless, the overlap between these two behaviours is such that, even if potential differences are taken into account, considerable overlap would likely persist [i.e. differences caused by, for example, surface effects were shown to decrease the distance covered in responses near the surface compared to those away from the surface by approximately 25% after 100 ms due to higher drag (Webb et al., 1991)].

Field observations of *H. littorale* also suggest that the performance of ABE in natural environments is comparable to that of ER observed in the laboratory, and that these performance levels are significantly higher than those observed in routine turns. Fast motion when gulping air makes particular sense in the field where ponds are typically highly turbid and fish become visible to aerial predators only when surfacing. It is likely that wild fish retain this behaviour in the laboratory, even in clear water, because of the unfamiliar environment and the perceived risk of predation normally associated with surfacing.

Because previous work has shown that escape responses can be triggered either by Mauthner cells or by slower acting parallel neurons (Eaton et al., 2001), the question remains as to whether the ABE observed here are controlled by Mauthner cells. Escape responses triggered by Mauthner cells tend to show shorter escape latencies than non-Mauthner cell responses (Eaton et al., 2001; Kohashi and Oda, 2008). Furthermore, lower turning rates were observed in escape responses of fish in which Mauthner cells and associated neurons had been ablated, compared to intact fish (Liu and Fetcho, 1999), suggesting that the Mauthner system is associated with fast turning rates. It can be hypothesised that, among all the ER we observed in the present study, the ones that are most likely to be Mauthner-cell mediated are those with the shortest escape latencies and the fastest turning rates. We found a significant relationship between escape latencies and turning rate, such that ER with short latencies also showed fast turning rates, as previously observed in other species of teleosts (Domenici and Batty, 1994; Domenici and Batty, 1997). We considered the ER with ≤12.5 ms escape latencies as good candidates to be Mauthner-cell responses, based on Eaton et al. (Eaton et al., 2001) who found the latencies in Mauthner cell and non-Mauthner cell responses in goldfish (*Carassius auratus*) to be 12.6 and 17.1 ms, respectively. In *H. littorale*, the range of turning rates (TR_max_) of these short-latency ER (2686–4215 degrees s^−1^) overlaps with the range of turning rate values observed in ABE (942–4840 degrees s^−1^), suggesting at least some of the ABE recorded here are likely to be controlled by Mauthner cells, although conclusive evidence would need Mauthner cell recordings during the turn. Furthermore, although potentially *H. littorale* may be able to anticipate the need to turn, it is also possible that the fish cannot precisely determine where the surface is while swimming vertically, and therefore they may need to generate a sudden turn (hence using a short latency such as that provided by Mauthner cells) as soon as the surface is perceived (possibly by barbels, see below) to avoid emerging excessively.

In terms of what may trigger the Mauthner cell (or the parallel neurons), it is possible that air-breathing events in *H. littorale* may be controlled by spontaneous activation (rather than external stimulation) of the Mauthner cells. Spontaneous (voluntary) activation of Mauthner cells has been suggested to play a role in post-feeding turns (Canfield and Rose, 1993) and object-striking C-bends observed in goldfish (Canfield, 2007). However, it is also possible that the timing of ABE may be regulated by contact with the surface. Catfish possess very sensitive barbels (Hoagland, 1933; Caprio, 1975) and contact of chemoreceptors located on these barbels with the air above the water surface, especially in the turbid water in which catfish live, may be used for precise timing of the C-bend contraction which re-directs the fish towards the bottom. Interestingly, other species of catfish [*Corydoras aeneus* (Kramer and McClure, 1980) and *Plecostomus punctatus* (Gradwell, 1971)] have been observed to show a similar behaviour whereby the fish follow their air-breath with a dash towards the bottom. However, their air-breathing kinematics have not been compared with that of escape responses; hence it would be worthwhile testing if our findings also apply to other species of air-breathing catfish.

An additional functional explanation for the fast motion performed during air-breathing in *H. littorale* may be related to one or more of the possible functions of air-breathing. In *H. littorale*, and in similar species of catfish, air-breathing may provide a number of functions in addition to respiration per se, especially when performed in normoxic conditions. For example, air in the intestine is also a requirement for buoyancy (Gee and Graham, 1978) and such a function was suggested for another species of catfish (*Corydoras aeneus*) that makes quick dashes to the surface in normoxia (Kramer and McClure, 1980). In addition, air-breathing can facilitate the passage of food through the digestive tract (Persaud et al., 2006) and can increase hearing capabilities in catfish because of the connection with the inner ear via the Weberian ossicles (Lechner and Ladich, 2008). Because our observations were carried out in normoxia, ABE may also have been related to such functions in addition to providing air for the respiratory organ. Furthermore, we observed the release of bubbles of air from the anus at the end of the ABE (supplementary material Fig. S1). This has previously been observed in other air-breathers respiring across sections of the gut (Persaud et al., 2006) and results from displacement of previously inhaled air situated within the intestine with freshly inhaled air. It is possible that the fast body C-bend may facilitate such air expulsion by quickly reducing the space of the body cavity with high pressure from the axial muscles. The fast release of air from the anus may in turn facilitate the unidirectional air flow in the respiratory intestine to transport digesta through the intestine quickly (Persaud et al., 2006). This mechanism would allow the respiratory intestine to be clear of digesta and function as an efficient gas exchange organ without being disrupted for long periods (Persaud et al., 2006).

Regardless of the specific adaptive value of using C-starts during ABE, the current results clearly suggest that similar kinematics may be employed in behaviourally diverse contexts and with or without external stimulation, i.e. from anti-predator responses to gulping air at the surface. The possibility that fish may be able to perform a C-start whenever the context demands an extremely rapid movement highlights the flexibility of the neuro-motor control of fast-swimming motions in fish.

## Supplementary Material

Supplementary Material
