# Supplementary material for: Fast-starting after a breath: air-breathing motions are kinematically similar to escape responses in the catfish Hoplosternum littorale
Source: Biol Open. 2014 Dec 19;4(1):79–85. doi: 10.1242/bio.20149332 (PMC4295168; doi:10.1242/bio.20149332)
Supplement: Supplementary Material [file supp_4_1_79__index.html]

Fast-starting after a breath: air-breathing motions are kinematically similar to escape responses in the catfish Hoplosternum littorale — Fast-starting after a breath: air-breathing motions are kinematically similar to escape responses in the catfish Hoplosternum littorale — Supplementary Material 

# Fast-starting after a breath: air-breathing motions are kinematically similar to escape responses in the catfish *Hoplosternum littorale*

## bio.20149332 Supplementary Material

**Files in this Data Supplement:**

- Supplementary Material - Paolo Domenici et al. doi: 10.1242/bio.20149332
