# Supplementary material for: Fast-starting after a breath: air-breathing motions are kinematically similar to escape responses in the catfish Hoplosternum littorale
Source: Biol Open. 2014 Dec 19;4(1):79–85. doi: 10.1242/bio.20149332 (PMC4295168; doi:10.1242/bio.20149332)
Supplement: Supplementary Material [file supp_bio.20149332_bio.20149332-s1.pdf]

Biology Open (2015) 000, 1–7 doi:10.1242/bio.20149332

Supplementary Material
Paolo Domenici et al. doi: 10.1242/bio.20149332

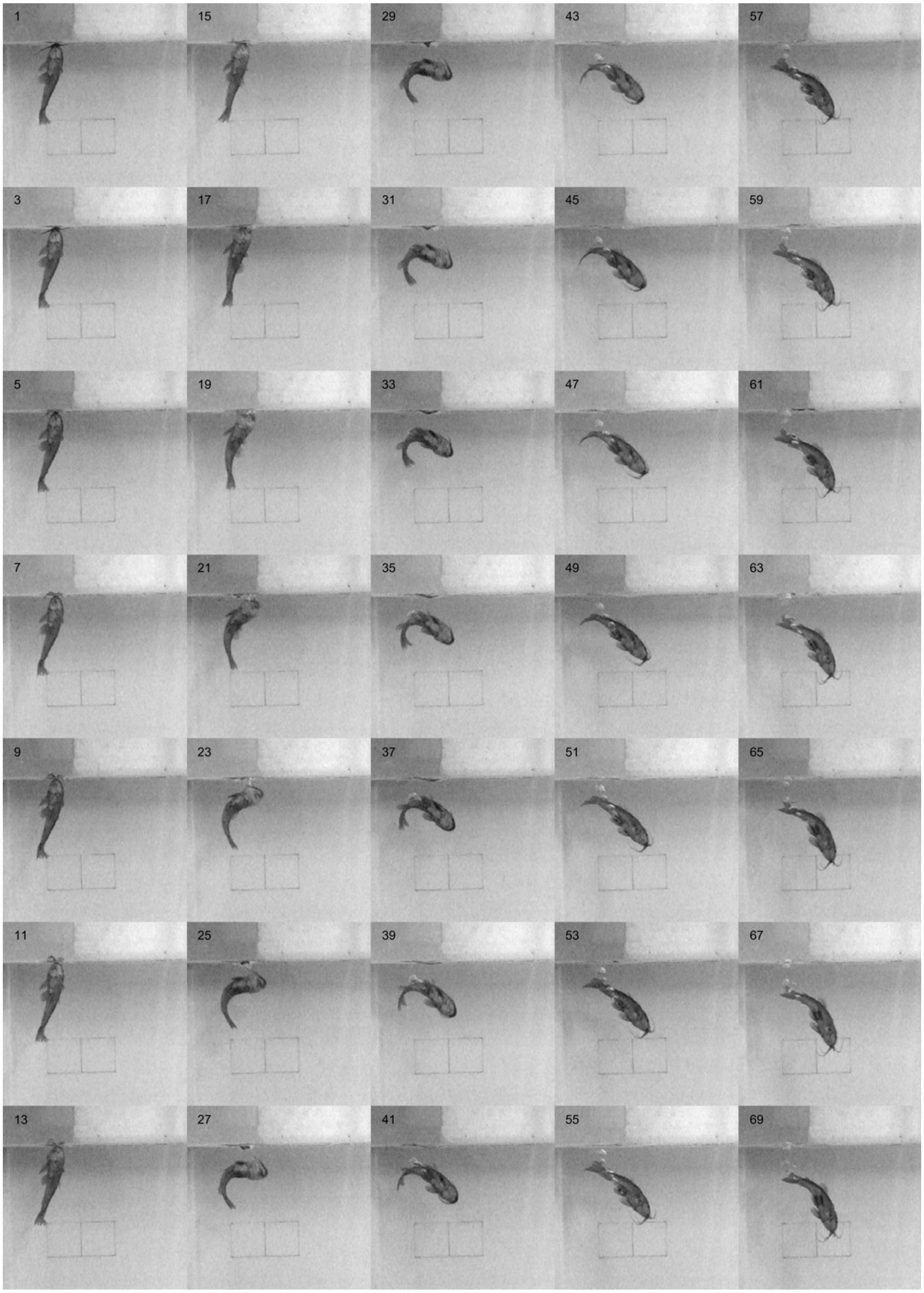

Fig. S1. Example of an air-breath performed by *Hoplosternum littorale* in the laboratory set-up. This example shows every second frame (time interval 8.33 ms) of a high-speed video recording, from the fish approaching the water surface to descending towards the bottom of the aquarium after the air-breath. Note the expulsion of air from the anus (frame 29 and onwards) during the post-air-gulping C-bend. Square markings on back wall are 5 × 5 cm.

Biology Open (2015) 000, 1–7 doi:10.1242/bio.20149332

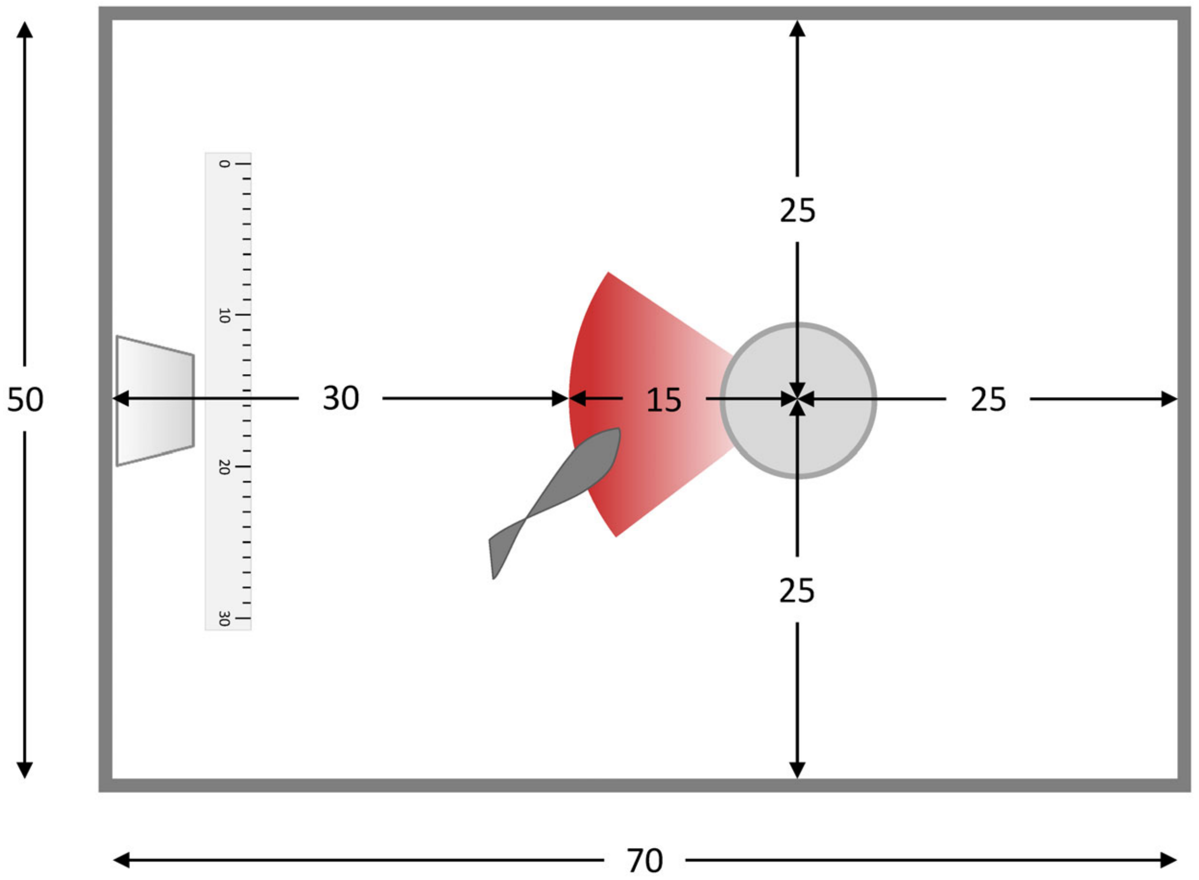

Fig. S2. Top view of the tank used for the escape response experiments. The circle indicates the tube within which the stimulus was released. Release of the stimulus occurred when the fish entered the drop zone from the front (i.e. when the fish entered the red shaded area from the circular side; not present in the original setup). The trapezoid on the left indicates the mirror. The ruler was placed on the bottom of the tank and served as reference for later analyses. Measurements are in cm. Scale is approximately 1:5.
